# Increasing the Chemical Variety of Small-Molecule-Based TLR4 Modulators: An Overview

**DOI:** 10.3389/fimmu.2020.01210

**Published:** 2020-07-10

**Authors:** Alessio Romerio, Francesco Peri

**Affiliations:** Department of Biotechnology and Biosciences, University of Milano-Bicocca, Milan, Italy

**Keywords:** TLR4—Toll-like receptor 4, medicinal chemistry, inflammation, drug development, endotoxin

## Abstract

Toll-Like Receptor 4 (TLR4) is one of the receptors of innate immunity. It is activated by Pathogen- and Damage-Associated Molecular Patterns (PAMPs and DAMPs) and triggers pro-inflammatory responses that belong to the repertoire of innate immune responses, consequently protecting against infectious challenges and boosting adaptive immunity. Mild TLR4 stimulation by non-toxic molecules resembling its natural agonist (lipid A) provided efficient vaccine adjuvants. The non-toxic TLR4 agonist monophosphoryl lipid A (MPLA) has been approved for clinical use. This suggests the development of other TLR4 agonists as adjuvants or drugs for cancer immunotherapy. TLR4 excessive activation by a Gram-negative bacteria lipopolysaccharide (LPS) leads to sepsis, while TLR4 stimulation by DAMPs is a common mechanism in several inflammatory and autoimmune diseases. TLR4 inhibition by small molecules and antibodies could therefore provide access to innovative therapeutics targeting sepsis as well as acute and chronic inflammations. The potential use of TLR4 antagonists as anti-inflammatory drugs with unique selectivity and a new mechanism of action compared to corticosteroids or other non-steroid anti-inflammatory drugs fueled the search for compounds of natural or synthetic origin able to block or inhibit TLR4 activation and signaling. The wide spectrum of clinical settings to which TLR4 inhibitors can be applied include autoimmune diseases (rheumatoid arthritis, inflammatory bowel diseases), vascular inflammation, neuroinflammations, and neurodegenerative diseases. The last advances (from 2017) in TLR4 activation or inhibition by small molecules (molecular weight <2 kDa) are reviewed here. Studies on pre-clinical validation of new chemical entities (drug hits) on cellular or animal models as well as new clinical studies on previously developed TLR4 modulators are reported. Innovative TLR4 modulators discovered by computer-assisted drug design and an artificial intelligence approach are described. Some “old” TLR4 agonists or antagonists such as MPLA or Eritoran are under study for repositioning in different pharmacological contexts. The mechanism of action of the molecules and the level of TLR4 involvement in their biological activity are critically discussed.

## Introduction

The immune system is a complex molecular and cellular machinery evolved to defend a multicellular organism from external pathogens and internal damages. It consists of innate immunity, based on the recognition of microbial pathogen-associated molecular patterns, PAMPs, and endogenous danger-associated molecular patterns, DAMPs, and adaptive immunity, mediated by the generation of a wide collection of antigenic sensors—the antibodies, produced by B cells (1).

Innate immunity is the first line of defense of a multicellular organism against internal or external threats. The molecular sensors of innate immunity are pattern recognition receptors (PRR), a large protein category comprising C-type Lectin Receptors, NOD-like receptors, RIG-I-Like Receptors and, most importantly, Toll-like Receptors. Toll-like Receptors (TLRs) are a family of proteins; in humans, 10 TLRs have been identified that recognize different molecular determinants or patterns from bacteria, viruses, and fungi (2).

**Figure 1:**
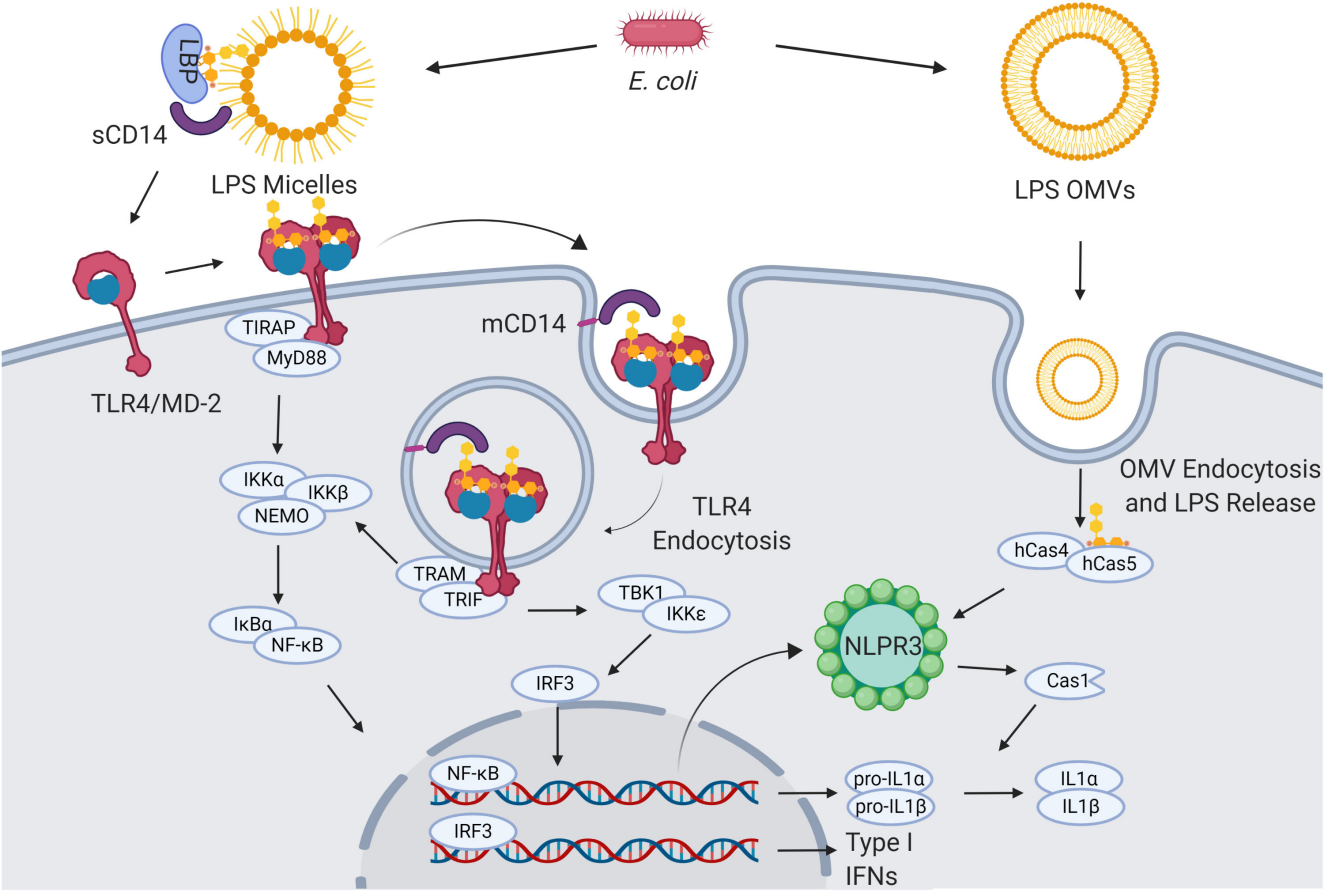
LPS signaling. Extracellular gram-negative bacteria release LPS in the form of micelles or OMVs. OMVs and micelles containing LPS can be delivered intracellularly where LPS activates caspase-dependent responses (right). Soluble LPS-binding protein (LBP) allows CD14 to capture LPS monomers. CD14 increases the sensitivity of TLR4-MD2 for LPS and favors the re-location of the complex formed by LPS, CD14, and TLR4-MD2 in the plasma membrane lipids rafts. Once in the lipid rafts, TLR4-MD2 starts TIRAP-MyD88-dependent responses. CD14 also induces the endocytosis of LPS and TLR4-MD2. From endosomes TLR4-MD2 triggers the TRAM-TRIF pathway and thereby sustains the activation of NF-κB and the production of type I IFNs (5–8).

TLR4, found in the plasma membrane of neutrophils, macrophages, dendritic and endothelial cells, selectively recognizes and responds to Gram-negative bacteria lipopolysaccharide (LPS) and lipooligosaccharide (LOS) (3, 4) (Figure 1).

**Figure 2:**
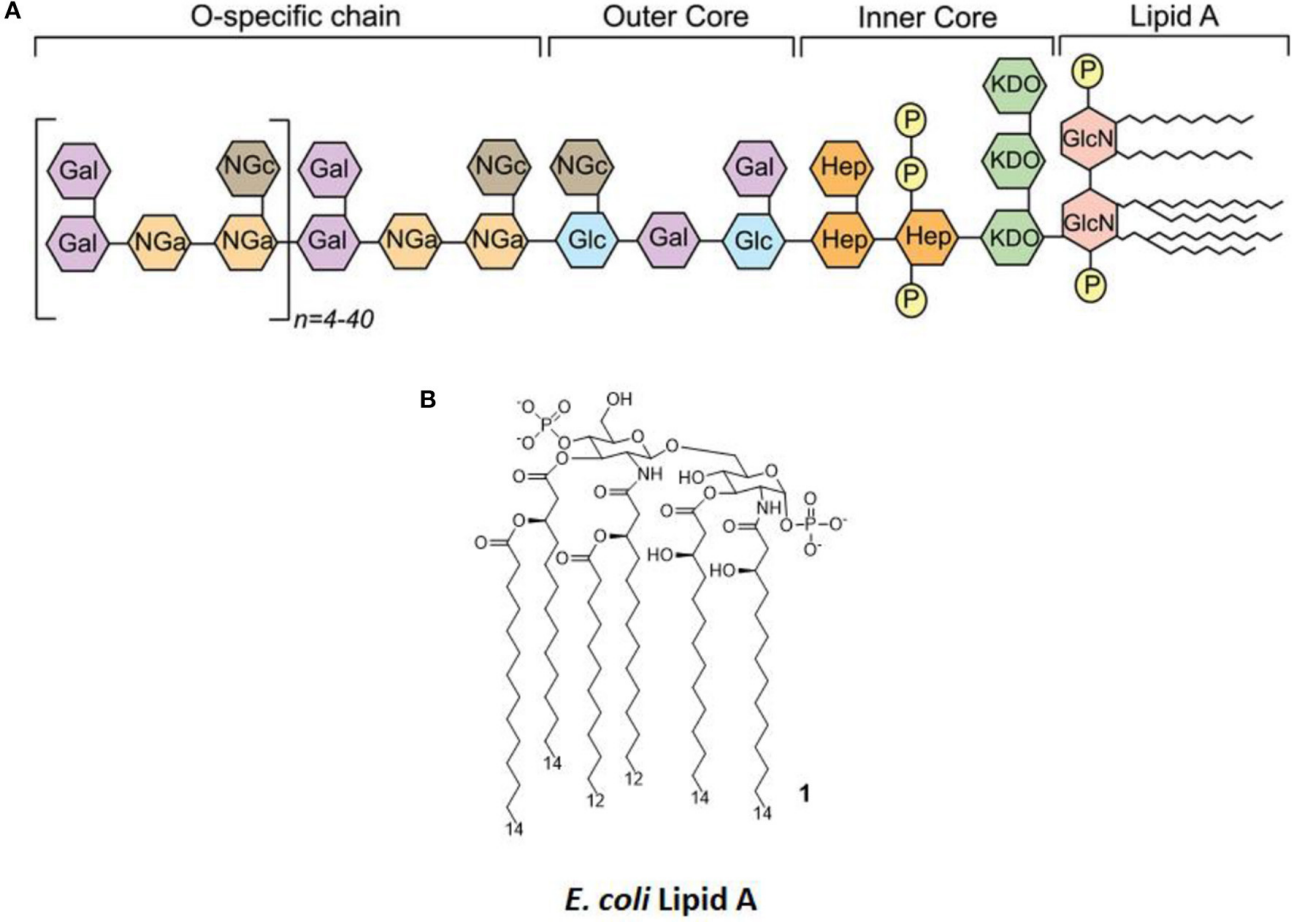
LPS Structure. **(A)** General structure, highlighting the different regions of LPS (O-antigen, core, and lipid A) and the various sugars composing it. **(B)** The molecular structure of *Escherichia coli's* lipid A.

LPS (Figure 2A) is the main chemical component of the Gram-negative bacteria outer membrane, and its chemical structure is characterized by a polysaccharide, the O-antigen, and a shorter oligosaccharide, the core region, bound to a glycolipid moiety called lipid A. Lipid A (Figure 2B) is the minimal LPS portion required to trigger immune activity through binding of CD14 and subsequent binding to the TLR4/MD-2 dimer on the plasma membrane (9, 10).

LPS is released from bacterial membrane as micelles or can be actively secreted via the formation of outer membrane vesicles (OMVs) (11). OMVs can directly deliver LPS in the cytosol of immune cells, where inflammatory caspases (caspase-4/5) serve as a specialized LPS receptor to induce the activation of the inflammasome and the production of bioactive interleukin-1β (IL-1β) and IL-18 (5).

In contrast to OMVs, the LPS contained in micelles requires the presence of accessory soluble proteins, such as LPS-binding protein (LBP), and, subsequently, CD14 and MD-2 must be recognized by TLR4.

LBP is required for transferring LPS monomers from micelles to TLR4-MD2 via the interaction with both soluble and membrane-anchored CD14 (6, 12, 13). The interactions between LBP and CD14 form a “capture and concentration module” upstream of TLR4-MD2 that regulates the ligand availability. The process starts with the contact of LPS micelles with a soluble LPS-binding protein (LPB). CD14 is then recruited, and a transient ternary complex (LPS micelle-LBP-CD14) is formed. LPS transfer happens during this phase in which, via electrostatic interactions, LBP catalyzes multiple rounds of LPS monomer transfer to either soluble or membrane-bound CD14 (sCD14 and mCD14, respectively). Subsequently, s/mCD14 dissociates from the complex, and the single LPS molecule bound to the CD14 is then transferred to MD2 with the assistance of LRR13-LRR15 domains of TLR4 that trigger the dimerization of TLR4-MD2 and its activation (6, 13). Concomitantly with LPS presentation, mCD14 also facilitates the relocation of TLR4-MD2 in lipid rafts, where multiple signaling molecules are recruited to contribute to cell activation (14). Lipid rafts also favor the action of TLR4-independent effectors, such as specialized proteins for the internalization of the complex formed by LPS, mCD14, and TLR4-MD2 (7, 15). Once engaged by CD14, TLR4-MD2 undergoes an internalization process and moves into the endosomal compartment, where it triggers the TRIF-Related Adaptor Molecule (TRAM) and TIR-Domain-Containing Adapter-Inducing Interferon-β (TRIF)-dependent pathway, which sustains the activation of NF-κB and also induces the production of type I interferons (IFNs).

TLR4 excessive activation by LPS can lead to pathologies such as sepsis and septic shock, one of the leading death causes in western world, with a mortality rate between 20 and 50%; furthermore, it can induce the immune system to attack cells from its own organism, causing and array of autoimmune diseases (16, 17).

Modulating TLR4 activation and signaling is therefore of fundamental importance from a pharmacological and clinical point of view. On one hand, innate immunity stimulation is useful for the development of vaccine adjuvants or cancer immunotherapeutic drugs (1, 18). On the other hand, TLR4 inhibition is a therapeutic approach to Gram-negative and sterile sepsis as well as autoimmune inflammatory pathologies such as atherosclerosis, rheumatoid arthritis, or hemorrhagic shock (15, 19–21). Indeed, two compounds, Eritoran and Tak-242, reached phase III clinical trials as antisepsis agents, and both failed to meet their endpoints (21, 22).

In the perspective of developing new TLR4-directed drugs, the recent achievements (last 3 years, from 2017) on the discovery of synthetic and natural molecules that modulate TLR4 activity as agonists or antagonists are reviewed as a follow-up of our recent review on this topic (23). We focus on small molecules with drug-like properties, dividing them in two main categories according to their chemical structure, namely glycolipid- and non-glycolipid-based TLR4 modulators (Tables 1, 2).

**Table 1:** TLR4 agonists presented in this review, ranked by chemical structure (glycolipid or non-glycolipid) and stage of drug development.

**Compound**	**Class**	**Drug Development Stage**
MPLA (24)	Glycolipid based	Approved by FDA as vaccine adjuvant
BECC438 (25)	Glycolipid based	*In vitro*
GLA (26)	Glycolipid based	Clinical
LAM (27)	Glycolipid based	*In vitro*
E6020 (28)	Non-glycolipid	*In vivo*
1Z105 (29)	Non-glycolipid	*In vivo*
PTC (30)	Non-glycolipid	*In vitro*
LS-like (31)	Non-glycolipid	*In vitro*
VS1-like (32)	Non-glycolipid	*In vitro*
Saturated cardiolipins (33)	Non-glycolipid	*In vitro*

*All agonists are validated according to the three postulates described in the text (34)*.

**Table 2:** TLR4 antagonists presented in this review, ranked by chemical structure (glycolipid or non-glycolipid), stage of drug development, and mechanism of action (MOA).

**Compound**	**Class**	**Drug Development Stage**	**MOA**
FP7-like (35, 36)	Glycolipid based	*In vivo*	Competitive inhibition
LAM (37)	Glycolipid based	*In vitro*	Competitive inhibition
IAXO (38)	Glycolipid based	*In vivo*	Competitive inhibition; LPS sequestration
TAK-242 (39)	Non-glycolipid	Clinical	Non-competitive inhibition
Calixarenes (40)	Non-glycolipid	*In vitro*	Competitive inhibition
Opioid (41)	Non-glycolipid	*In vitro*	Competitive inhibition
Pip2 (42)	Non-glycolipid	*In vivo*	Competitive inhibition
Unsaturated cardiolipins (33)	Non-glycolipid	*In vitro*	Competitive inhibition
Alpinetin (43)	Non-glycolipid	*In vivo*	Down-regulation of TLR4 expression
Ferulic acid (44)	Non-glycolipid	*In vivo*	TLR4/MD-2 complex disruption

The validation of a new chemical entity as a selective TLR4 agonist or antagonist is a crucial step in the drug development process. While TLR4 antagonist (inhibitor) validation is straightforward, as the TLR4 selectivity can be assessed through competition experiments with LPS -the natural TLR4 agonist-, TLR4 agonism assessment requires more careful investigation because it could be affected by false positive results due to endotoxin contamination. Therefore, three postulates have been proposed in order to ascertain and validate TLR4 agonists activity: (I) the requirement of bothTLR4 and MD-2 for the agonist effect; (II) the agonist or the active portion of it should be reproduced synthetically, and the synthetic derivative should preserve TLR4 activity; and (III) a specific molecular interaction between the agonist and TLR4/MD-2 must be identified (34).

Glycolipid-based compounds are Lipid A mimetics that can be obtained by (1) chemical modification of natural LPS/Lipid A, (2) direct extraction of lipid A variants after bacterial engineering, or (3) full chemical synthesis (45–47).

Some non-glycolipid compounds still reproduce the arrangement of lipid chains and phosphates found in the Lipid A but are devoid of the disaccharide scaffold (as in the case of Eisai's E6020). Others have a chemical structure totally unrelated to lipid A and have been developed by computer-assisted drug design (CADD) and a machine learning approach or have been selected from libraries of compounds (29, 32, 48).

The clinical and pharmacological potential of newly discovered, low-molecular weight (<2 kDa) compounds together with the preclinical and clinical validation level of known lead compounds is reviewed, paying special attention to validation of TLR4 targeting. Tables 1, 2 give a general picture of the state of the art in the clinical development of small-molecule-based TLR4 agonists and antagonists, respectively.

## Glycolipid-Based TLR4 Modulators

### Agonists

#### MPLA

**Figure 3:**
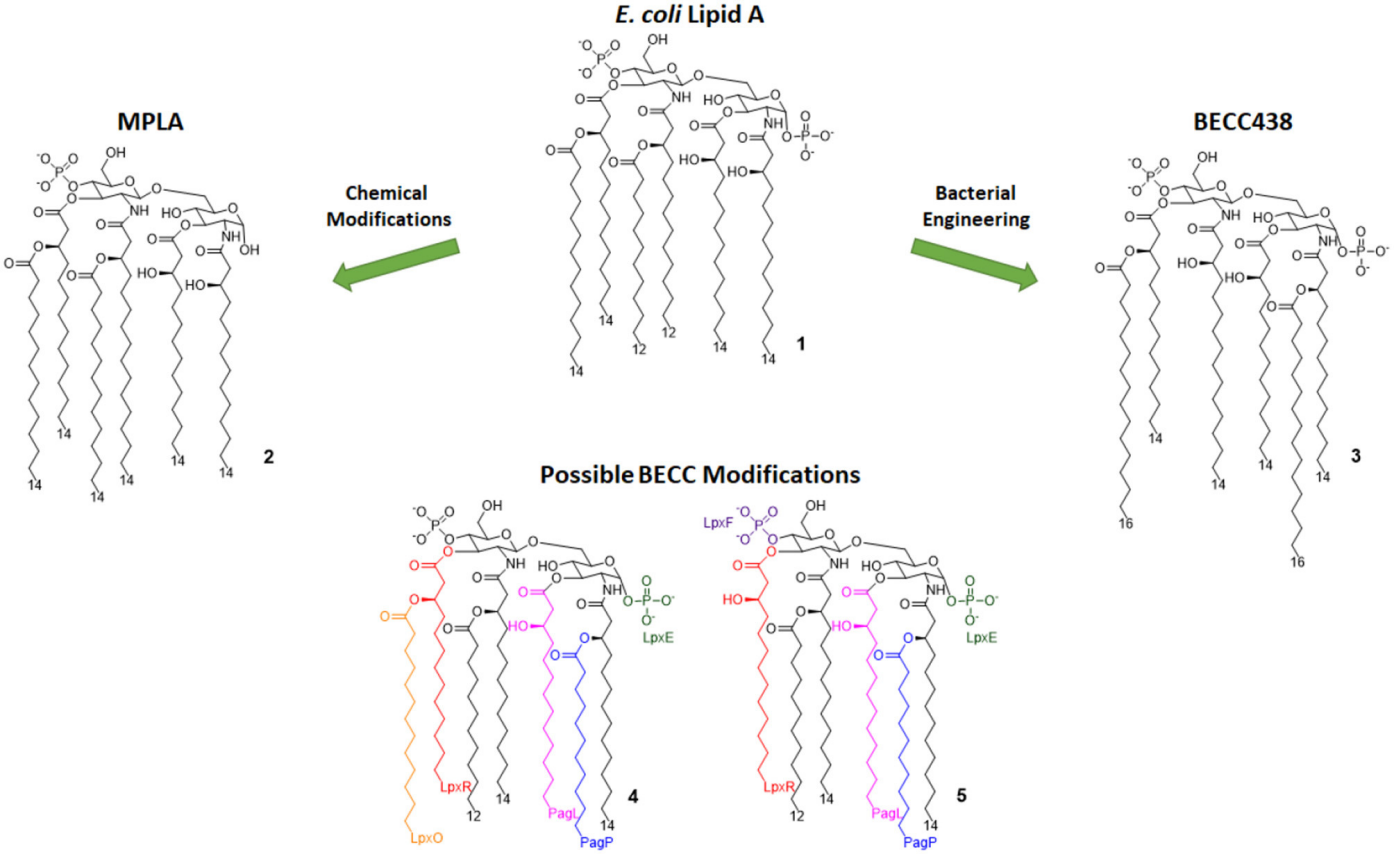
Lipid A variants obtained by chemical and enzymatic modification. **MPLA** is obtained by chemical hydrolysis of the C-1 phosphate of the Salmonella Minnesota LPS. **BECC** (Bacterial Enzymatic Combinatorial Chemistry) allows for the selective modification of single or multiple fatty acid chains or phosphates (depicted by colors associated to involved enzymes) in order to obtain compounds such as **BECC438** with high purity.

Monophosphoryl Lipid A (MPLA, compound 2, Figure 3) is a well-characterized TLR4 agonist (45). MPLA is chemically derived from *Salmonella minnesota* LPS through treatment with mild acidic conditions, as this achieves the cleavage of the lipid A portion from the oligosaccharide core and the hydrolysis of the 1-phosphate group. TLR4 requirement for MPLA action has been thoroughly validated by numerous studies involving TLR4 ^−/−^ mice (45, 49).

MPLA is a potent TLR4 agonist, but it is weaker than LPS, as MPLA's affinity to TLR4/MD-2 is weaker than LPS. It has been also suggested that MPLA-activated TLR4 signal goes only or preferentially through TRIF-dependent and not through MyD88-dependent cascade. TRIF bias has been proposed to be related to the weaker inflammatory power and the reduced toxicity compared to LPS. TRIF bias also switches T-cell immunity to T_H_1 helper, better suited for long-lasting immunization (50, 51).

MPLA is the only TLR4 agonist to be approved by the FDA for the use as a vaccine adjuvant on human (Cervarix®, Fendrix®) (52, 53).

Because of its immunostimulating activity and the lack of toxicity, the use of MPLA has been envisaged in a wide array of clinical settings. In a recent study it has been hypothesized that MPLA stimulatory activity on the innate immune system could mitigate the radiation injury provoked by ionizing radiation (IR) in cancer radiotherapy (24). Pre-treatment with MPLA prevented IR-provoked cell apoptosis *in vitro* and effectively attenuated tissue damage *in vivo*. Authors used siRNAs to knock down TRIF and MyD88 in wild type RAW264.7 cells. It was found that MPLA significantly inhibited apoptosis in TRIF knock-down cells, whereas, in MyD88 knock-down cells, MPLA had no effect on cell apoptosis induced by irradiation. These data point out that the MyD88 signaling pathway mainly accounts for the radioprotective effects of MPLA, which is in contrast to the TRIF-biased action of MPLA previously discussed.

#### Enzymatically Modified Lipid A

The approval of MPLA fostered the development of synthetic or semi-synthetic Lipid A variants as TLR4 modulator candidates for clinical use.

In 2013, Needham et al. developed a new technology to obtain naturally derived TLR4 agonist by using a technique that was recently named bacterial enzymatic combinatorial chemistry (BECC). BECC consists in bacterial gene engineering, removing or adding enzymes in LPS biosynthesis pathway, allowing the isolation of LPS/lipid A variants with non-natural modifications and their straightforward isolation from bacterial pellets without further purification (54).

In 2017, BECC was performed on an attenuated *Yersinia pestis* strain, consequently obtaining lipid A variants that were then screened *in vitro* and *ex vivo*, showing TLR4 activation levels comparable to those obtained with previously described MPLA (46).

Particularly, a compound named BECC438 (compound 3, Figure 3) showed good *in vitro* activity, which suggested a follow-up study *in vivo* to confirm its viability as a vaccine adjuvant and to compare its efficacy to other adjuvants (Alum and PHAD). All mice immunized using non-formulated BECC438 as an adjuvant survived after being challenged with *Y. pestis*: indeed, BECC438 group's survival rate (100%) was better than both Alum and Glucopyranosyl Lipid Adjuvant (GLA, see next paragraph) groups (both scored 80% survival rate), suggesting that properly formulated BECC438 could exceed GLA efficacy and encouraging follow-up studies on its use as a vaccine adjuvant (25).

#### Glucopyranosyl Lipid Adjuvant (GLA)

**Figure 4:**
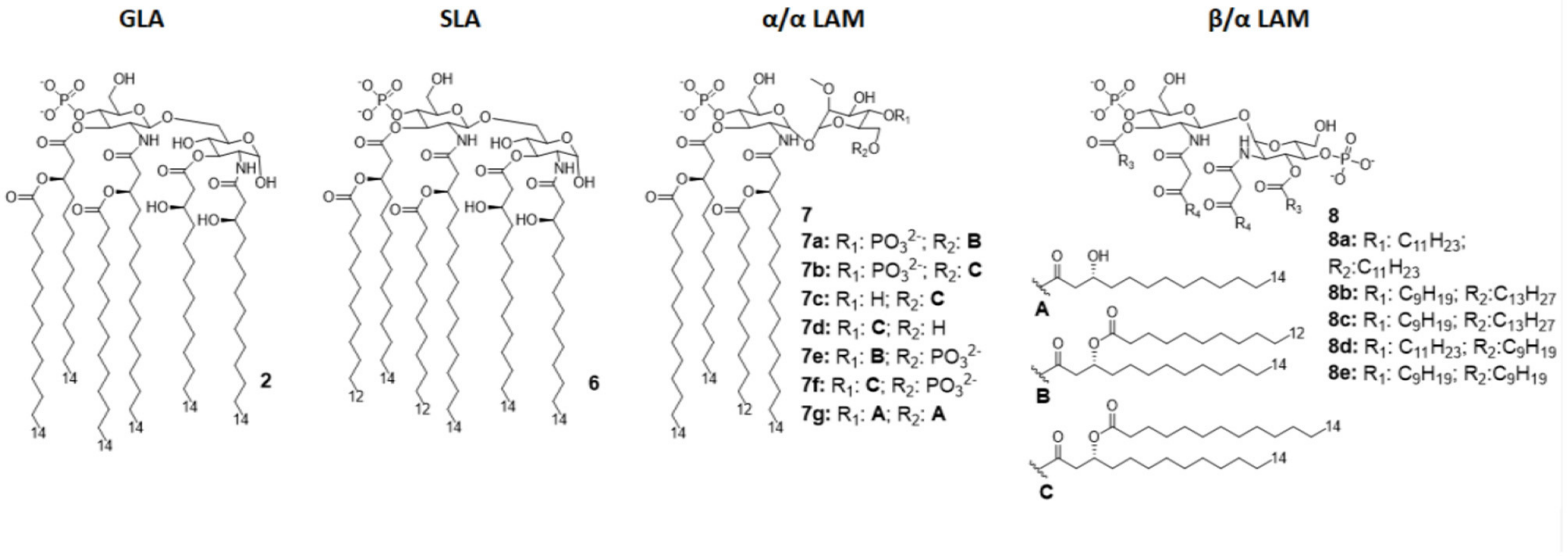
Disaccharide-based synthetic Lipid A analogs. **GLA** is a fully synthetic MPLA. **SLA** is a second generation GLA optimized to be more compatible with MD-2 (by introducing C12 acyl chains). **LAMs** are trehalose-derived compound: changing the absolute configuration of the glycoside bond LAM allow the switch from TLR4 agonism to antagonism.

The Glucopyranosyl Lipid Adjuvant (GLA, Figure 4) has been developed by Avanti Polar Lipids Inc. as a fully synthetic MPLA analog with TLR4 agonistic activity (tradename: phosphorylated hexa-acyl disaccharide PHAD®) (55, 56). Being fully synthetic, the main advantage of this compound is its chemical homogeneity, which improves activity and safety with respect to MPLA, a semi-synthetic molecule. Moreover, LPS contamination is avoided (57).

In recent years, GLA has been formulated as a vaccine adjuvant both as aqueous formulation (GLA-AF) and as an oil-in-water stable emulsion (GLA-SE). When compared to MPLA in terms of activity, it showed an overall better response (57, 58).

GLA-AF was tested as a nasal vaccine adjuvant for HIV immunization *in vivo* on mice and rabbits, resulting in a good immunization profile with strong mucosal immune responses (59, 60). In 2018, Anderson et al. tested HIV immunization in humans following nasal administration of a vaccine containing GLA-AF as adjuvant and the HIV-1 CN54gp140 antigen. Early transcriptional signatures were investigated to identify differentially expressed genes (DEG) and blood transcription modules (BTM) correlated with vaccination and successful immunization (26). Results were encouraging, indicating the activation of numerous vaccine related DEG and BTM, and this therefore suggests that immunization occurred. However, the small number of subjects involved and lack of analysis in the first 7 days suggest that additional studies are needed to validate data.

Recent advancement in cancer immunotherapy involving TLR to (re-)activate immune cells suggested the use of a GLA-SE (named G100) as a stand-alone cancer immunotherapeutic (61, 62).

Following a successful *in vivo* study on an A20 lymphoma murine tumor model in which half of the mice got regression in a dose dependent manner (63), a first clinical trial was started on a small number (n=10) of patients affected by merkel cell carcinoma (MCC), based on intratumoral injection of a low dose of G100. Out of the 10 patients, three presented local disease and were then treated with surgery (cohort A), while some presented a metastatic disease (cohort B). All patients in cohort A successfully completed surgery and radiotherapy after administration of G100, and two of them remained recurrence free; patients in cohort B received only G100 and two of them went in full remission (64).

The brilliant results obtained by GLA experimentations urged the development of an even better TLR4 glycolipid agonist, having higher efficiency and lower toxicity. In this way, Carter et al. recently developed a second-generation lipid adjuvant (SLA), reducing the length of two lipid chains from C14 to C12 (compound 6, Figure 4). Computational docking studies show that the reduction of the Hydrophobic part make this lipid A derivative better accommodate into the MD-2 hydrophobic pocket, allowing for and stronger interaction with TLR4/MD2 (65).

The activity of SLA and its TLR4 selectivity has been assessed both *in vitro, ex vivo*, and *in vivo* (65) SLA has been then formulated as an oil-in-water stable emulsion (SLA-SE) and tested *in vivo* as an adjuvant for nasal *Enterotoxigenic E. coli* vaccine in comparison with double-mutant LT (dmLT) adjuvant: results suggest that SLA-SE is at least as effective as dmLT, but it is able to further augment some of the specific immune responses (66).

#### Trehalose Derivatives (LAM)

While TLR4 plays a pivotal role in innate immunity, particularly protecting against infectious challenges and boosting adaptive immunity, it is not the only factor causing inflammation in the only LPS receptor. Indeed, caspase 4/5/11-mediated NLRP3 inflammasomes, activated by cytosolic LPS, is a crucial pathogenic factor in a variety of acute and chronic immune related diseases (67).

In order to obtain new agonists with increased TLR4/inflammasome selectivity, Zamyatina et al. aimed to design molecules capable of activating only the TLR4 pathway without activating NLRP3. To achieve this result, according to a computational structural analysis of the TLR4 dimerization process, two separate hydrophobic clusters are needed in the ligand to optimize the binding with the hydrophobic pocket of MD-2/TLR4, crosslinking the second MD-2^*^/TLR4^*^ and consequently forming the activated (TLR4/MD-2/ligand)_2_ complex. Seven novel trehalose-derived disaccharides were projected and synthesized based on an α,α-(1-1')-linked diglucosamine scaffold (Lipid A Mimetics, α/α LAMs, Figure 4). The conformational rigidity of the α,α glycosidic bond was exploited by rational design to obtain the two separate hydrophobic clusters for MD-2 binding and TLR4 activation (27, 37).

The activity of α/α LAM was tested on mononuclear cells (MNC), human airway epithelial cells (Calu-3) and human monocytic cell line THP-1 and observed that, while 4'-6-diphosphate compounds (compounds 7a-b and 7e-f, Figure 4) induced both TLR4 and caspase 4/11 activity, monophosphate compounds 7c and 7d (Figure 4) effectively decoupled TLR4 and NLRP3, exclusively activating TLR4 without triggering a NLRP3-dependent response: these results open the way for future synthesis of safer TLR4 agonists and for clarifying the role of caspase 4/11 activation in inflammasome (27).

Interestingly, changing the stereochemistry of α/α glycosidic bond into β/α bond, a shift from TLR4 agonism to antagonism was observed (37). Indeed, five novel β(1-1')α linked diglucosamine LAMs, containing 2-N-, 2'-N-linked β-ketoacyl lipid chains were synthesized (α/β-LAMs, compounds 8, Figure 4). These new compounds were then tested for their antagonist activity *in vitro*, obtaining full inhibition of LPS-stimulated cytokine production at 1 μg/mL concentration. Surprisingly, concentrations higher than 10 μg/mL showed reduced antagonist activity, probably because the formation of aggregates. Finally, molecular dynamics simulations showed that MD-2 affinity of LAMs is higher than LPS. The keto-enolic tautomerism on acyl chains of LAMs very likely provides free hydroxyls that can be involved in additional interactions through hydrogen bonds with residues at the rim of MD-2 binding pocket (37).

### Antagonists

#### Anionic Monosaccharide-Based TLR4 Antagonists

Synthetic monosaccharide mimetics of Lipid X, a monosaccharide biosynthetic precursor of lipid A, showed TLR4 antagonist activity in murine macrophages (68, 69).

A large panel of synthetic monosaccharide-based TLR4 modulators, named Gifu Lipid As, contain one or two phosphates groups and a variety of modifications in fatty chains length and nature as well as in their binding mode to glucosamine: esters, amide, ethers, and amines were used (70, 71).

Following this trend, monosaccharide-based pure TLR4 antagonists, called FP compounds, were developed, and they are active in inhibiting the LPS-stimulated TLR4-dependent cytokine production in human and murine macrophages in a dose-dependent manner (IC_50_ from 0.46 to 3.2 μM) (72).

FP compounds were tested as potential therapeutics in different clinical settings. The lead compound FP7, with two C-14 fatty acid chains, showed the ability to protect motoneurons from microglia activated by LPS in an *in vitro* motoneurons/microglia co-culture model of ALS (73).

The capacity of FP7 to protect mice from DAMP/TLR4 activation as a consequence of influenza virus pulmonary infection was evaluated (74). FP7 turned out to protect mice from acute lung injury (ALI), one of the most prominent influenza-related damages, and increase survival after viral infection with an efficiency similar to Eritoran, a well-established TLR4 antagonist developed by Eisai (75). In this model of infection, ALI would induce DAMP release from damaged tissues, likely HMGB1 and oxidized phospholipids, which, in turn, hyperactivate TLR4 with a subsequent cytokine storm and acute sepsis-like syndrome. An experiment on DCs activated by HMGB1 suggested that FP7 can block HMGB1-dependent TLR4 activation. Further data should be collected to assess the activity of this type of antagonist to block TLR4 activation by oxidized phospholipids (oxPL) and other DAMPs that highly likely are produced by ALI.

TLR4 gene deletion in hematopoietic and non-hematopoietic cells protects animals against cardiovascular diseases (CVD), suggesting a key role of the receptor in these pathologies (76). The potential of FP molecules to impact on inflammatory CVD was investigated *in vivo* on Angiotensin II-infused apolipoprotein E-deficient mice. After validating the capacity of FP7 to inhibit cytokine production *in vitro* on human umbilical vein endothelial cells (HUVEC), THP-1, and RAW 264.7 cells, *in vivo* experimentation demonstrated that Angiotensin II and FP7 co-administration prevented the initiation of sterile inflammation, protecting mice from consequent CVD (77).

Interestingly, in addition to inhibition of LPS-induced TLR4 signaling, FP7 negatively regulated TLR4 activation in response to ligands of sterile inflammation, namely, hydroperoxide-rich oxidized LDL (oxLDL) *in vitro* and Angiotensin II infusion *in vivo* (77).

Taken together, these studies suggest that FP molecules are able to contrast the action of structurally diverse DAMPs from HMGB1 (74) to oxLDL (77).

The synergistic action of FP monosaccharides with antibacterial peptides neutralizing LPS was recently investigated (78). After LPS stimulation, FP7 was co-administrated to cells together with two anti-microbial peptides: cecropin A–melittin (CA–M) or LL-37, a human cathelicidin that binds to and neutralize LPS (79, 80). A synergy between TLR4 antagonists and cationic peptides was observed in inhibiting TLR4-dependent cytokine production and NF-kB activation. Interestingly, the synergy was observed also in a case where TLR4 was activated with lectins. DOSY NMR experiments and TEM microscopy images suggest a change in the supramolecular aggregation state of peptides caused by the interaction with FP7 (78).

**Figure 5:**
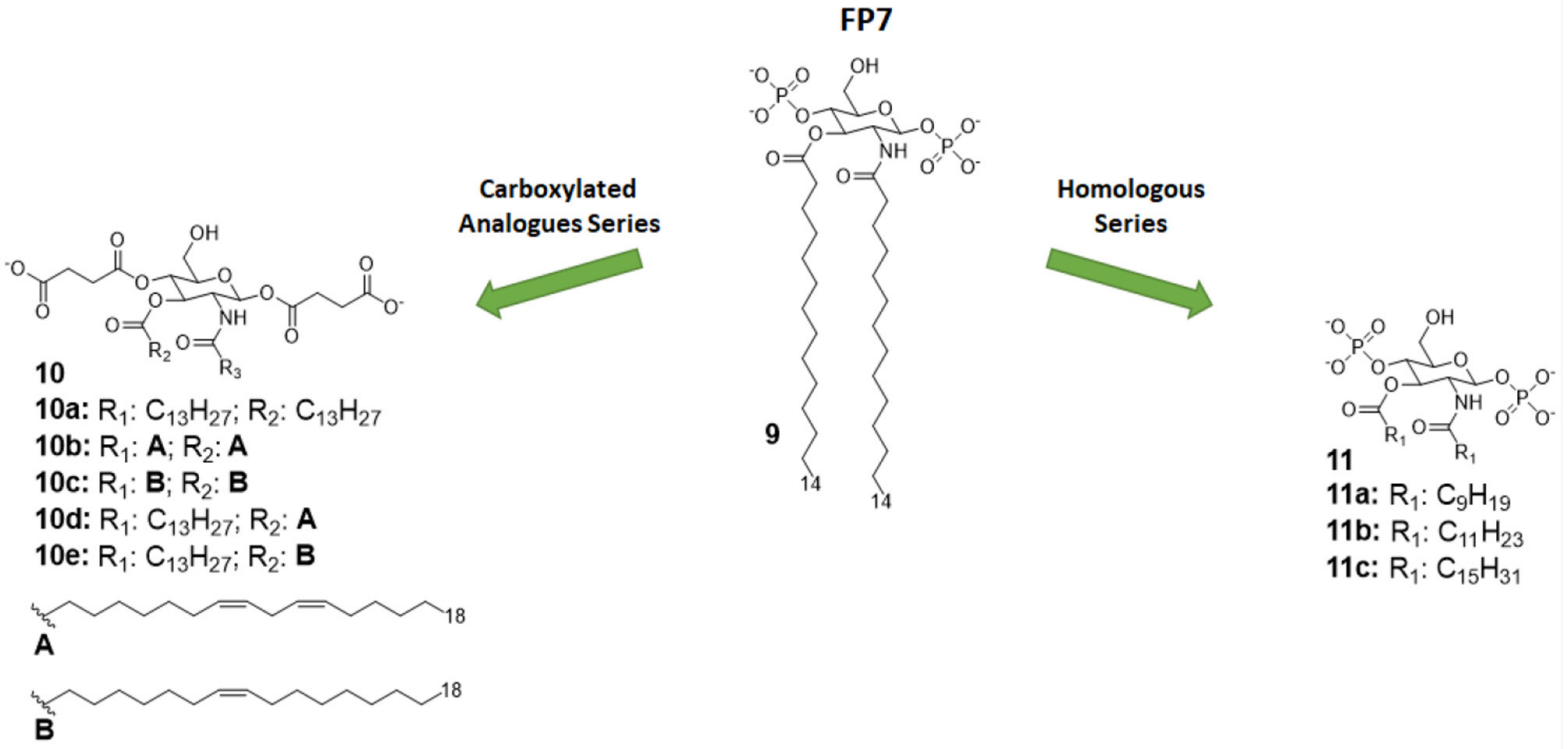
Monosaccharide TLR4 antagonist of the FP series and their carboxylate analogs.

Two studies focused on the investigation of the structure-activity relationship (SAR) in FP monosaccharides (as depicted in Figure 5): one explored the effect of the length of saturated fatty on the TLR4 activity and the second investigated both the effect of unsaturated fatty chains and the suitability of succinate groups as bioisosteres of phosphate groups (35, 36).

In both studies, molecules were firstly designed *in silico* through docking with MD-2 receptor followed by and molecular dynamics simulation. Molecules were then synthetized and tested for their capacity to bind to MD-2 and to inhibit LPS-stimulated TLR4 activation in human and murine macrophages. The first study pointed out a very precise trend of activity on cells and MD-2 binding potency, indicating the compounds with C12 and C14 carbon chains (Figure 5) are the most active in inhibiting TLR4 activation and cytokine production (35). Interestingly, the compound with C16 was found to be totally inactive. The C12 and C14 compounds (compounds 11b and 9, respectively, Figure 5), named FP12 and FP7, respectively, were shown to form less tight aggregates with a higher fluidity of fatty acid chains than the C16 compound. As in the case of lipid A derivatives, it is very likely in this class of amphiphilic monosaccharides that the supramolecular structure and the stability of aggregates influences the biological activity (8, 81).

The carboxylate analogs (Compounds 10, Figure 5) with two succinate esters units instead of phosphates retained TLR4 antagonist activity with an IC_50_ in the same range of lead compound FP7. Furthermore, the structure of the fatty acid chains turned out to be essential to TLR4 activity. Paralleling the SAR results in the FP family, the series of saturated fatty chains presented a maximum activity again around C12 and C14, while shorter (C10) and longer (C16) chains were unable to interact with MD-2 to inhibit TLR4-dependent cytokine production. On the other hand, unsaturated lipids retained activity even with longer chains (C18). It is known that unsaturated fatty acids are present in TLR4 antagonists such as the LPS synthesized by *Rhodobacter sphaeroides* (RS-LPS) or *Rhodobacter capsulatus* (RC-LPS) and the synthetic Eritoran. The results reported in this paper confirm that the presence of one unsaturation in the fatty acid chains favors the switch to antagonism (36).

#### Cationic Monosaccharide-Based TLR4 Antagonists

**Figure 6:**
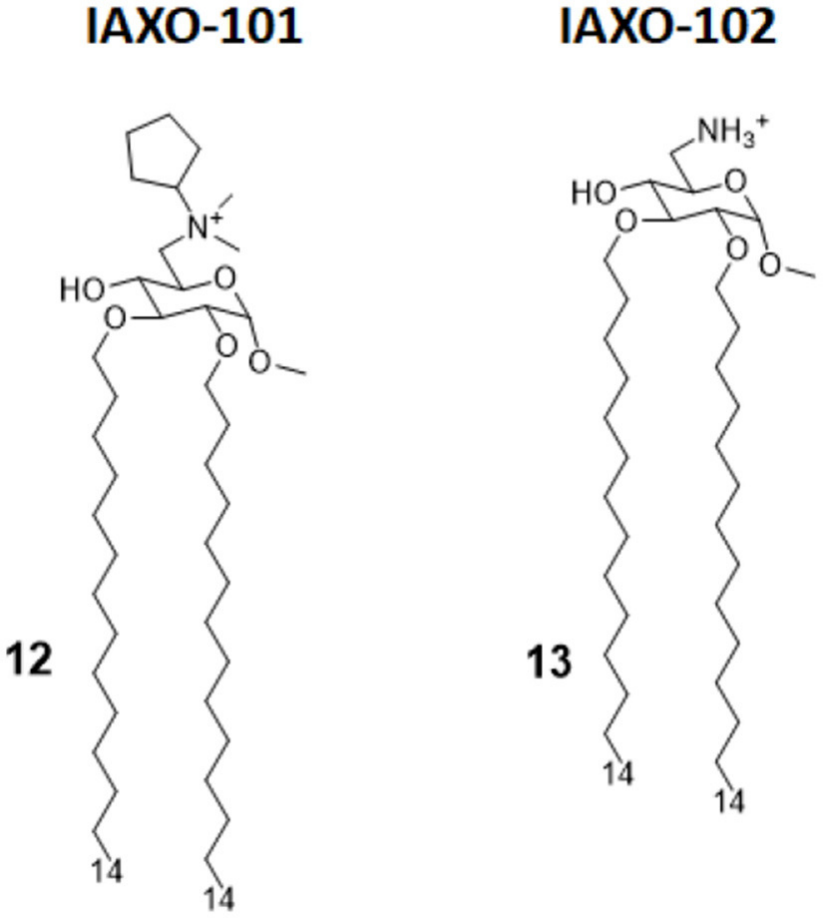
IAXO-101 and IAXO-102 are glycolipid compounds bearing a positive charge. They form stable aggregates with LPS and compete with LPS for TLR4 binding.

IAXO compounds are a class of cationic amphiphiles active as TLR4 antagonists. They are formed by a glucopyranose or a benzylamine core linked to two C14 lipid chains through ether bonds (compounds 12 and 13, Figure 6) (38).

IAXO's TLR4 antagonism is very likely the combination of two effects: in the form of cationic liposomes, these molecules form stable co-aggregates with LPS and make it less available for binding with CD14 and MD-2 (82). On the other hand, mechanism studies clearly show the ability of IAXOs to bind CD14 and MD-2, competing with LPS and displacing it from receptors (83–85).

In a new study on the role of TLRs in Placental Malaria (PM) by Barboza et al., IAXO 101 was used to assess the involvement of TLR4 in infant morbidity and mortality in a group of pregnant mice affected by *Plasmodium berghei* NK65^GFP^, and its effect was compared with a group of TLR4^−/−^ mice. While TLR4^−/−^ mice did not show PM, and their fetuses did not show differences in body weight compared to non-infected WT mice, experiments demonstrated that mice treated with IAXO 101 2 weeks after infection showed a partial reverse in placental malaria, and their fetuses had an intermediate body weight between infected and non-infected WT mice. In addition to demonstrating the involvement of TLR4 in PM, this study also highlights the viability of IAXO 101 as a treatment for this pathology, which causes high neonatal mortality (86).

Another recent application of IAXOs has been the prevention of blood–brain barrier (BBB) disruption after subarachnoid hemorrhage (SAH). Okada et al. aimed to study the linkage between TLR4 activation and inflammatory BBB disruption (39). In an animal study, SAH was induced in C57BL/6 male mice, which were eventually treated with two different dosages of IAXO 102 (compound 13, Figure 6) after 30 min. This resulted in a significantly improved neurological score and in clear protection from BBB disruption. A control experiment was conducted involving TAK-242, a well-established TLR4 antagonist, providing similar results. Those experiments highlighted for the first time that BBB disruption after SAH is linked to TLR4 activation and can be efficiently reversed by administration of potent TLR4 antagonists as a treatment for post-SAH BBB disruption (39).

## Non-Glycolipid TLR4 Modulators

### Agonists

#### Linear Lipid A Analogs (E6020)

**Figure 7:**
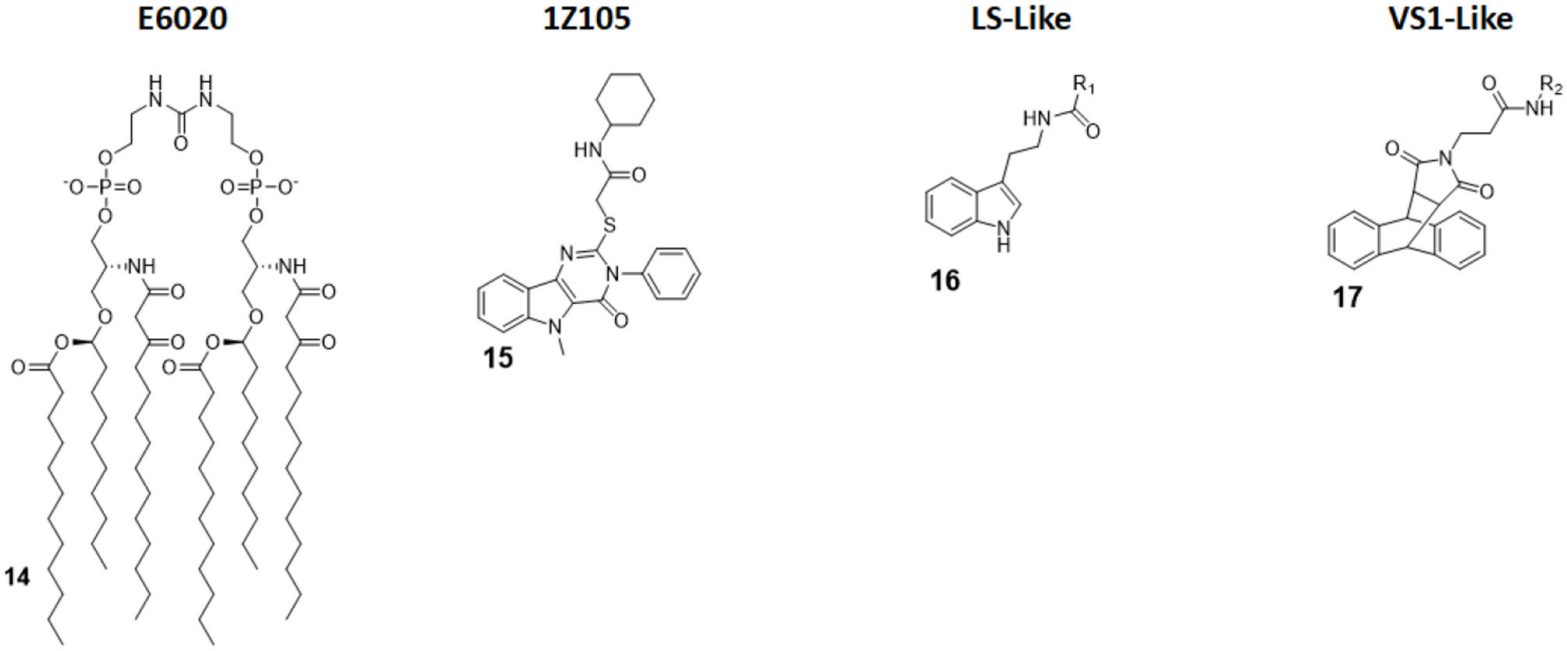
Non-Glycolipid TLR4 agonists. **E6020** is a linear lipid A analog developed by Eisai Inc., currently in clinical trial in cancer immunotherapy. **1Z105**, a pyrimidoindole, is currently being tested *in vivo* as vaccine adjuvant. **LS-** and **VS1-**like compounds are novel TLR4 agonist structures obtained by computational approach.

E6020 (compound 14, Figure 7) is a synthetic agonist patented by Eisai Inc., which has been previously been experimented on as a vaccine adjuvant *in vivo*, and it turned out to be a viable alternative to traditional alum adjuvant both on boosting mucosal and systemic antibodies responses and in enhancing vaccine efficacy on a toxic shock syndrome model (48, 87, 88).

Following these successes, it has been recently assessed on the central nervous system (CNS) to test its activity in enhancing remyelination in spinal cord white matter following lysolecithin-induced demyelination. Remyelination is mediated by oligodendrocytes, which are vulnerable to a series of pathologies and infection: when their number is low, they can be replaced by oligodendrocyte progenitor cells (OPCs) after differentiation. However, myelin debris prevents OPCs differentiation, effectively hindering remyelination process. Indeed, it seems that the presence of E6020 stimulates macrophages to remove myelin debris, which is a vital step; this allows for and enhances remyelination in lysolecithin-induced demyelination animal models. The remyelination is therefore linked to TLR4 activation. This novel study opens the possibility to use TLR4 agonists to repair damages caused by aging or injury, and this prevents a series of CNS pathologies, including dementia (28).

#### Pyrimidoindoles

Pyrimido[5,4-b]indoles are a class of synthetic TLR4 agonists first identified by Cottam and coworkers through a high-throughput screening (HTS) approach (89). Subsequently, a structure-activity relationship (SAR) study allowed to select 1Z105 (compound 15, Figure 7) as the best agonist compound. 1Z105 has been tested as a vaccine adjuvant in combination with 1V270, a TLR7 agonist (89, 90).

As a follow-up of these studies, an influenza vaccine formulated with both 1Z105 and 1V270 was shown to function *in vivo* through TLR4 and TLR7 activation without any significant off-target effect, and it succeed in inducing protective immunity. The activation of TLR4 by 1Z105 mainly activated the MyD88 pathway. Furthermore, the TLR4 and TLR7 agonists worked synergistically to reach a high adjuvant potency, allowing for a dose reduction of the antigen to achieve equivalent protection and enhancing the vaccine safety profile (29).

#### New Rationally Designed TLR4 Agonists

Michaeli et al. recently projected linear and cyclic peptides with the ability to bind MD-2/TLR4 and CD14/TLR4 by computer-assisted drug design (CADD). They used *ab initio* methods coupled with machine learning discovery software, which allowed the finding of a higher percentage of active molecule compared with an HTS approach. New cyclic peptide sequence containing also D-amino acids to increase conformational rigidity and drug-likeness were designed to dock with hMD-2 and the N-terminal region of h-CD14 using the CYCPEP program (30). Subsequently, *in silico* designed MD-2 and CD14 ligand peptides were synthesized and tested for their activity under physiologically relevant conditions by determining IL-1β release upon culture in human whole blood. Out of 27 linear and 26 cyclic peptides, two peptides (PTC-A-40 and PTC-A-83) were shown to be active in stimulating IL-1β production, validating the use of *ab initio* method to search for TLR4 ligands (30).

Honegr et al. investigated the advantages of *in silico* drug design in the search for TLR4 agonists, by using Ligand- or Structure-Based Virtual Screening (LBVS or SBVS). A large library of molecules (130,000) was screened *in silico* for their capacity to bind to a 3D model of hTLR4/MD2 heterodimer (PDB ID: 4G8A, RCSB Protein Data Bank). Two hit compounds were identified that optimized binding score: a N-(2-(1H-indol-3-yl)ethyl)benzamide (LS-like, compound 16 Figure 7) and a anthracene-succinimide hybrid (VS1-like, compound 17, Figure 7). Both compounds were then synthetized and chemically modified for SAR studies. While LS and LS-derived molecules didn't achieve a good activity profile (10% of MPLA activity), VS1 and VS1-derived molecules showed a much more promising efficacy when tested *in vitro* and *ex vivo*, scoring 50% of MPLA activation (31, 32).

### TLR4 Antagonists

#### TAK-242

**Figure 8:**
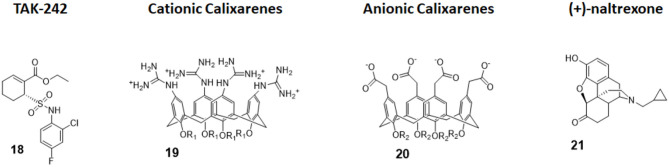
Non-glycolipidic TLR4 antagonists. **TAK-242** is a potent TLR4 inhibitor non-competitive toward LPS. Cationic Calixarenes, were able to block TLR4 signaling while anionic calixarenes were inactive. **(+)-N17**-subsituted Naltrexone derivatives are the only known TLR4 antagonists able to cross the BBB to date and are drug candidate for neuropathic pain.

TAK-242 (compound 18, Figure 8) is a cyclohexene carboxylic ester derivative, produced by Takeda Pharmaceutical Company Ltd, that shows strong action (IC_50_ 1 to 11 nM) as a specific TLR4 non-competitive inhibitor (91). Indeed, studies performed by Takashima et al. (92) and Matsunaga et al. (93) demonstrated that TAK-242 binds intracellularly TLR4: it acts as a Michael acceptor for Cys747 residue present in the TIR domain of TLR4. Therefore, TAK-242 disrupts the TIR domain conformation and subsequent interaction with both TIRAP and TRIF, and this inhibits both MyD88-dependent and MyD88-independent pathways (92, 93).

TAK-242 was administered on sepsis patients in various intensive care units worldwide in a phase III clinical trial. Unfortunately, trials were terminated because TAK-242 was ineffective in reducing mortality and in suppressing cytokines production. Although the reasons for failure are unknown, a combination of individual differences in severity of illnesses and delay in administration of the drug are thought to be the main causes. Furthermore, enrolment in the study of patients without Gram-negative bacteria infections probably affected the results (22).

A new study by Wang et al. sought to investigate TAK-242 influence in coronary microembolization (CME)-caused myocardial apoptosis, starting by the fact that TLR4 had been demonstrated to be a promising target for atherosclerotic cardiovascular diseases treatment (94). In the study, the authors were able to reproduce i*n vivo* models of CME in mice. CME mice treated with TAK-242 showed a significant improvement in cardiac function and a decrease in micro-infarction area and in apoptotic index when compared with untreated mice, validating TLR4 as a target in this pathology and suggesting treatment with TLR4 inhibitors as an efficient therapeutic approach. However, authors claim the necessity of further studies, as they only experimented short-term effects of TAK-242 and the animal model of CME, obtained by plastic microspheres injection, does not completely mimic microembolization in patients (95).

#### Calixarene Amphiphiles

Calix[4]arenes are cup-shaped organic molecules formed by four or more phenol units linked together by methylene bridges. Calix[4]arenes possess a central hydrophobic conical cavity, and both cavity rims could be chemically functionalized to improve or modulate water solubility. The presence of a cavity and the possibility to synthetically change their chemical structure and therefore modulate water solubility make calix[4]arenes, together with cyclodextrins and cucurbituryls, optimal hosts to carry small molecules and drugs. In recent years, there was a growing interest in calixarenes as drug carriers as they are biocompatible and show low cytotoxicity (96–99).

The capacity of amphiphilic calixarenes to modulate TLR4 signal was studied in cationic calix[4]arenes functionalized with guanidine groups on the upper rim (compounds 19, Figure 8) and anionic calix[4]arenes with carboxyl groups (compounds 20, Figure 8) (40).

Surprisingly enough, anionic calix[4]arenes, which should better mimic the negatively charged, amphiphilic lipid A, did not show any activity on TLR4. On the other hand, positively charged guanidinocalixarenes (compounds 19, Figure 8) successfully inhibited TLR4 activity in a dose-dependent manner, with an IC_50_ ranging from 0.7 to 63 μM. A previous report by Chen et al. (100) described the capacity of similar guanidino calix[4]arenes to neutralize the action of LPS by binding it. The authors sought therefore to verify if the activity of calixarenes in blocking TLR4 signals derived exclusively from LPS binding and neutralization or from a direct action on the TLR4/MD-2 complex or a combination of these two effects. Cells were treated with a plant lectin, which is known to activate TLR4 by a mechanism different than LPS, and then with different doses of guanidinocalixarenes. A dose-dependent TLR4 inhibition was still observed, and this is suggestive of a direct effect of calixarenes on the receptor (40).

#### Opioid Derivatives

The opioid inactive isomer (+)-naltrexone has emerged as the only known TLR4 antagonist having the required LogP to easily cross the blood–brain barrier, making it an interesting lead for the treatment of neuropathic pain and drug addiction (101). While a previous study by Wang et al. confirmed that (+)-naltrexone inhibits TRIF/TRAM pathway and binds to MD-2, the molecular mechanism of action and the precise binding to TLR4/MD-2 and/or CD14 interaction is still unclear (102).

Wang et al. recently investigated the interaction with MD-2 by molecular docking and experimentally validated the found binding affinity by *in vitro* fluorescence binding studies. Studying a variety of (+)-naltrexones derivatives substituted with different groups on nitrogen N-17, it turned out that the enhancement of the hydrophobic character of the molecules by the introduction of octyl, phenylethyl, or methylcyclopropyl groups (compound 21, Figure 7) improved MD-2 binding affinity. Adding a methyl group onto N-17 leads to quaternary ammonium cations, which showed poor MD-2 binding affinity (K_d_ > 40 μM) and lost the TLR4 antagonistic activity. Authors concluded that the binding of (+)-naltrexone and its derivatives to MD-2 are primarily driven by hydrophobic interactions. However, polar interactions, which includes both electrostatic interactions and polar solvation free energy, were negatively correlated with experimentally determined binding affinities (41).

#### Peptide Antagonists PIP2 and cPIP2

A phage display (PD) library of 12-mer peptides was constructed by enriching through six rounds of biopanning against hTLR4. One of the five selected peptides, PIP2, a rather hydrophobic 12-mer, inhibited LPS-stimulated TNF-α and IL-6 production in murine and human macrophages with an IC_50_ of 40 μM (42). Besides the relatively weak activity, PIP2 showed some out-of-target inhibitory effects on TLR2. In order to assess the PIP2 mechanism of action, fluorescence binding studies, surface plasmon resonance, confocal microscopy with both fluorescently labeled TLR4, and peptide and molecular dynamics experiments were run. All experiments pointed to a direct interaction between PIP2 and MD-2. Encouraged by these promising results, the authors cyclized PIP2 by a lactam bridge (cPIP2), a common strategy to force α-helix and rigidify small peptides, with the aim to enhance activity and drug-likeness. Indeed, cPIP2 showed better inhibitory profile on TLR4 (IC_50_ 25 μM) and was further tested *in vivo* in rheumatoid arthritis (RA) mice model. cPIP2 successfully alleviated RA symptoms in mice over a period of 6 weeks, improving histological scores, which suggests the use of cyclic PIP2 as drug lead in RA (42).

#### Cardiolipin

Cardiolipins (CLs) is a family of tetra-acylated diphosphatidylglycerols naturally produced by animals, plants, bacteria and yeasts, and they have with different fatty chains lengths and saturations (33). Unsaturated CLs showed activity as TLR4 antagonists although the precise molecular mechanism remains to be studied (103, 104). As already mentioned in this review, unsaturated fatty acids are present in natural and synthetic TLR4 antagonist. This suggests that the unsaturation of the acyl chains contributes to enhance TLR4 antagonist behavior. An exhaustive SAR study on CL variants was recently published, and it focused on the influence of chain lengths and saturation degree (105).

**Figure 9:**
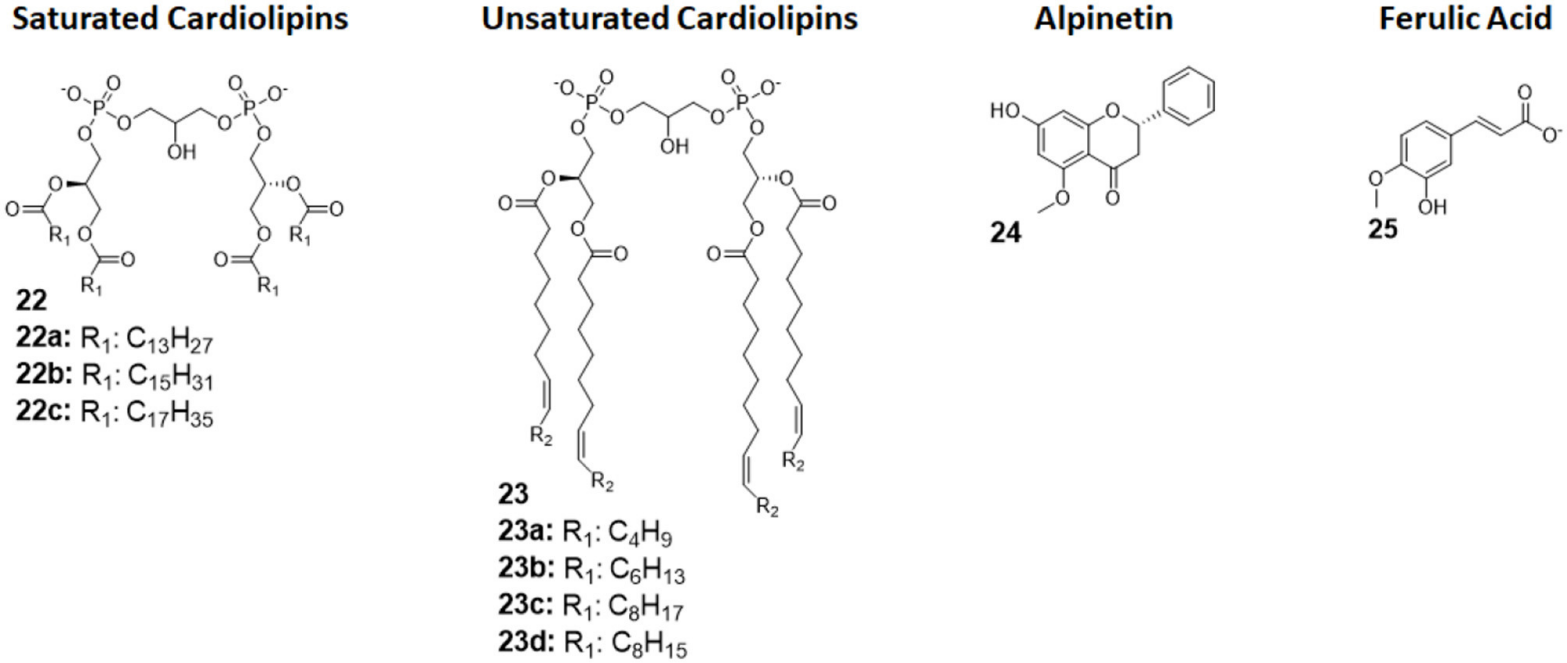
Cardiolipin derivatives 21 and 22 modulate TLR4 activity as agonists or antagonists. The switch between agonism and antagonism seems to be related to the presence of unsaturation on fatty chains. **Alpinetin** (45) is a natural flavonoid which can modulate TLR4 by downregulating its expression by the cell. **Ferulic Acid** (46) showed to antagonize TLR4 by disrupting the TLR4/MD-2 complex.

The activity on cells of a series of saturated and unsaturated derivatives (compounds 22 and 23, Figure 9) was tested. Results showed that all unsaturated CLs (compounds 23, Figure 9) are active as antagonists on human and murine TLR4 (in HEK-blue cells and murine macrophages), successfully inhibiting receptor signaling with IC_50_ ranging from high nM to low μM. Saturated CLs (compounds 22, Figure 9) can activate TLR4, inducing pro-inflammatory cytokines production (105). The only exception to this empirical rule is saturated C14:0 CL, which acted as agonist in murine cells but as antagonist in human cells, similarly to Lipid IVa (105, 106).

#### Alpinetin

Alpinetin (compound 24, Figure 9) is a natural flavonoid extracted from the plant *Alpinia katsumadai Hayata*. It has been demonstrated to possess anti-inflammatory activity, protecting against LPS-related damages both *in vitro* and *in vivo* (107). Subsequent studies clarified that alpinetin exerts its action as an agonist of PPAR-γ, which, in turn, downregulates TLR4 expression, effectively inhibiting receptor signaling (108): it is an indirect TLR4 antagonist. Recent studies proved alpinetin ability to protect mice against kidney damages and endometritis caused by LPS administration. Alpinetin-treated mice showed attenuated LPS-induced histopathological changes; furthermore, alpinetin was showed to inhibit pro-inflammatory cytokines secretion in a dose-dependent manner (43, 109).

#### Ferulic Acid

Ferulic Acid (compound 25, Figure 9) is a phenolic compound abundant in various herbs, fruits, and vegetables, and it is extracted from *Ligusticum wallichii*. It has been recently shown to have various properties, among which antioxidant and anti-inflammatory effects in murine cells, but its exact mechanism remained unclear (110).

Two recent studies claim that FA can protect against LPS-induced bovine endometritis *in vitro* and against LPS-induced acute kidney injury *in vivo* by suppressing NF-κB and MAPK signaling, which strongly point toward a TLR4-related mechanism of action (111, 112).

Indeed, Rehman et al., in a recent study in which they demonstrate FA positive effects against LPS-induced neuroinflammation in mice, were able to elucidate FA Activity. By *in silico* molecular docking, the authors reported that FA action is exerted by interfering with MD-2 binding site on TLR4, effectively disrupting the TLR4-MD-2 complex and preventing LPS recognition and formation of the activated dimer (TLR4/MD-2/LPS)_2_. However, the proposed mechanism, although intriguing, still lacks experimental proof, as it has only be postulated on the basis of molecular docking (44).

## Conclusions

We presented here last advancements in the field of TLR4 modulators, focusing on small molecules of both synthetic and natural origin, as a follow-up of recent reviews on this topic (23, 113).

All TLR4 modulators described in this review have been validated or at least evaluated for their capacity to interact specifically with TLR4 and MD-2. For most of the molecules molecular docking calculations and experimental binding studies are available to assess their mechanism of action based on the binding of TLR4/MD-2.

While glycolipid-based TLR4 modulators present a high degree of similarity between them, as they mimic lipid A chemical structure, non-glycolipid TLR4 modulators can have a variety of structures, ranging from smaller bicyclic compounds, as the antagonist TAK-242 was approved for clinical use, to larger calix[4]arenes or peptides.

The structural diversity leads inevitably to a diversity in effects, potency, and mode of actions, which are reflected in different pharmacodynamics.

TLR4 is the only TLR that initiates two different signal pathways: the MyD88 and the TRAM/TRIF, ending up with the production of inflammatory cytokines or type-I interferons.

Interestingly, TLR4 modulators with different chemical structures can activate differentially the two different pathways.

Glycolipid TLR4 agonists, such as MPLA (and its synthetic form GLA)- or BECC-derived compounds, were found to preferentially activate the TRIF way, and this skews lymphocytes toward a T_H_1 response, which is better suited to pathogens and pathogen-infected cells opsonization and elimination.

On the other hand, the pyrimidoindole derivative 1Z105 was found to activate TLR4 in a MyD88-biased fashion, leading to a T_H_2 response, which is better in fighting parasites and extracellular pathogens infections. This difference in the mechanism of action is critical and can be exploited to optimize the rational design of vaccine adjuvants since they could be more effectively formulated to elicit the most desirable response against a specific pathogen (25, 29, 45, 50, 57).

The structural diversity of TLR4 modulators leads therefore to different pharmacodynamics but also to different pharmacokinetic and targeting of different body districts.

Some glycolipid-based compounds, such as monosaccharidic FP7s and disaccharidic LAMs, can be effectively used systemically since they are water soluble and have a good distribution. On the other hand, highly hydrophobic non-glycolipid TLR4 antagonists, such as (+)-naltrexone, are better suited to target CNS diseases such as neuroinflammation, neuropathic pain, and neurodegenerative diseases including Alzheimer's disease (AD) (36–38, 41).

Another intriguing consequence of the structure diversity is the variety of the mechanism of actions. While agonists generally activate TLR4 by direct binding to MD-2, antagonists have much more possibilities to impact on TLR4 activity besides competitive inhibition.

Two interesting examples discussed within this review are TAK-242 and ferulic acid. Thanks to its cyclohexene carboxylic acid ester structure, TAK-242 acts as a good electrophilic Michael acceptor and is able to form a covalent bond with TLR4. This changes the TLR4 ectodomain conformation and modifies the conformation of TIR domain and the subsequent interaction with TRIFF and MyD88 (93). Ferulic acid binds in TLR4/MD-2 interaction interface. Consequently, this prevents TLR4/MD-2 complex formation, which, in turn, blocks the formation of the final activated complex (TLR4/MD-2/LPS)_2_ (44).

Structure refinement and optimization of MD-2 binding *in silico* has proven to be a viable technique in this field, as recently shown by the works of Honegr, Michaeli, and Achek reviewed here (30–32, 42).

Novel anti-inflammatory mechanisms indirectly acting on TLR4 have emerged in last years: namely, alpinetin's ability to downregulate TLR4 gene expression by enhancing PPAR-γ activity and the activity of LAMs in regulating non-canonical NLRP3 inflammasomes at the level of caspases 4/11 (27, 108).

The presence of cross-talk between different inflammation pathways, in particular between TLR4 signaling and the non-canonical inflammasome initiated by cytosolic LPS, suggest the use of a combination of small molecules to simultaneously block several pathways or, alternatively, the development of dual ligands able to bind to TLR4 and caspases (27, 37).

## Author Contributions

FP mainly contributed to abstract, introduction and tables. AR mainly contributed to figures, main body of the review and conclusion.

## Conflict of Interest

The authors declare that the research was conducted in the absence of any commercial or financial relationships that could be construed as a potential conflict of interest.
